# Supplementary material for: Bruxism associated with short sleep duration in children with autism spectrum disorder: The Japan Environment and Children’s Study
Source: PLoS One. 2024 Dec 6;19(12):e0313024. doi: 10.1371/journal.pone.0313024 (PMC11623795; doi:10.1371/journal.pone.0313024)
Supplement: S1 Text — (DOCX) [file pone.0313024.s001.docx]

STROBE Statement—checklist of items that should be included in reports of observational studies

|  | Item No. | Recommendation | Page  No. | Relevant text from manuscript |
| --- | --- | --- | --- | --- |
| **Title and abstract** | 1 | (*a*) Indicate the study’s design with a commonly used term in the title or the abstract | P3 | “from the Japan environment..” |
|  |  | (*b*) Provide in the abstract an informative and balanced summary of what was done and what was found | P3 | “Although bruxism is…”  “The odds ratio of the… ” |
| Introduction | | | |  |
| Background/rationale | 2 | Explain the scientific background and rationale for the investigation being reported | P5 | “Bruxism is an involuntary…” |
| Objectives | 3 | State specific objectives, including any prespecified hypotheses | P7 | “Thus, using a dataset from..” |
| Methods | | | |  |
| Study design | 4 | Present key elements of study design early in the paper | P7 | “The aim and procedure of…” |
| Setting | 5 | Describe the setting, locations, and relevant dates, including periods of recruitment, exposure, follow-up, and data collection | P8 | “Pregnant women were…” |
| Participants | 6 | (*a*) *Cohort study*—Give the eligibility criteria, and the sources and methods of selection of participants. Describe methods of follow-up  *Case-control study*—Give the eligibility criteria, and the sources and methods of case ascertainment and control selection. Give the rationale for the choice of cases and controls  *Cross-sectional study*—Give the eligibility criteria, and the sources and methods of selection of participants | P8-9 | “Pregnant women were recruited…”  “Of the 104,059 pregnancies…” |
|  |  | (*b*) *Cohort study*—For matched studies, give matching criteria and number of exposed and unexposed  *Case-control study*—For matched studies, give matching criteria and the number of controls per case | N/A |  |
| Variables | 7 | Clearly define all outcomes, exposures, predictors, potential confounders, and effect modifiers. Give diagnostic criteria, if applicable | P8-10 | “2.2 Occurrence of habitual…”  “2.3 Prevalence of ASD…” |
| Data sources/ measurement | 8* | For each variable of interest, give sources of data and details of methods of assessment (measurement). Describe comparability of assessment methods if there is more than one group | P7  P8-10 | “This study was based on…”  “2.2 Occurrence of habitual…” |
| Bias | 9 | Describe any efforts to address potential sources of bias | P17-18 | “Our study had several…” |
| Study size | 10 | Explain how the study size was arrived at | P7-8 | “Of the 104,059 pregnancies…” |

Continued on next page

| Quantitative variables | 11 | Explain how quantitative variables were handled in the analyses. If applicable, describe which groupings were chosen and why | P9-11 | “2.4 Sleep duration in early…”  “2.5 Covariates” |
| --- | --- | --- | --- | --- |
| Statistical methods | 12 | (*a*) Describe all statistical methods, including those used to control for confounding | P11 | “2.6 Statistical analysis…” |
|  |  | (*b*) Describe any methods used to examine subgroups and interactions | P11 | “Using the data, the participants ” |
|  |  | (*c*) Explain how missing data were addressed | P11 | “Of the 83,720 participants…” |
|  |  | (*d*) *Cohort study*—If applicable, explain how loss to follow-up was addressed  *Case-control study*—If applicable, explain how matching of cases and controls was addressed  *Cross-sectional study*—If applicable, describe analytical methods taking account of sampling strategy | P8 | “Of 104,059 fetuses enrolled…” |
|  |  | (*e*) Describe any sensitivity analyses | P12 | “With a focus on the association...” |
| Results | | | | |
| Participants | 13* | (a) Report numbers of individuals at each stage of study—eg numbers potentially eligible, examined for eligibility, confirmed eligible, included in the study, completing follow-up, and analysed | P13 | “3.1 Demographic characteristics of the study participants…” |
|  |  | (b) Give reasons for non-participation at each stage | N/A | N/A |
|  |  | (c) Consider use of a flow diagram | P8 | “Of the 104,059 pregnancies…” |
| Descriptive data | 14* | (a) Give characteristics of study participants (eg demographic, clinical, social) and information on exposures and potential confounders | P13 | “3.1 Demographic characteristics of the study participants…” |
|  |  | (b) Indicate number of participants with missing data for each variable of interest | N/A | N/A |
|  |  | (c) *Cohort study*—Summarise follow-up time (eg, average and total amount) | P7 | “This study was based on the…” |
| Outcome data | 15* | *Cohort study*—Report numbers of outcome events or summary measures over time | P13 | “The prevalence of ASD was…” |
|  |  | *Case-control study—*Report numbers in each exposure category, or summary measures of exposure |  |  |
|  |  | *Cross-sectional study—*Report numbers of outcome events or summary measures | P13 | “The prevalence of ASD was…” |
| Main results | 16 | (*a*) Give unadjusted estimates and, if applicable, confounder-adjusted estimates and their precision (eg, 95% confidence interval). Make clear which confounders were adjusted for and why they were included | P14 | “3.2 Bruxism behaviour…” |
|  |  | (*b*) Report category boundaries when continuous variables were categorized | P9 | “2.4 Sleep duration in early…” |
|  |  | (*c*) If relevant, consider translating estimates of relative risk into absolute risk for a meaningful time period | P14 | “The crude and adjusted ORs of ASD for habitual bruxism were…” |

Continued on next page

| Other analyses | 17 | Report other analyses done—eg analyses of subgroups and interactions, and sensitivity analyses | P14 | “3.3 Impact of sleep duration in…” |
| --- | --- | --- | --- | --- |
| Discussion | | | | |
| Key results | 18 | Summarise key results with reference to study objectives | P15 | “In this study, we analysed the…” |
| Limitations | 19 | Discuss limitations of the study, taking into account sources of potential bias or imprecision. Discuss both direction and magnitude of any potential bias | P17 | “Our study has several advantages and limitations. The JECS…” |
| Interpretation | 20 | Give a cautious overall interpretation of results considering objectives, limitations, multiplicity of analyses, results from similar studies, and other relevant evidence | P16 | “We found that the occurrence of habitual bruxism was higher…” |
| Generalisability | 21 | Discuss the generalisability (external validity) of the study results | P17 | “The JECS dataset used in our…” |
| Other information | |  | | |
| Funding | 22 | Give the source of funding and the role of the funders for the present study and, if applicable, for the original study on which the present article is based | P21 | “FUNDING INFORMATION  The Japan Environment and…” |

*Give information separately for cases and controls in case-control studies and, if applicable, for exposed and unexposed groups in cohort and cross-sectional studies.

**Note:** An Explanation and Elaboration article discusses each checklist item and gives methodological background and published examples of transparent reporting. The STROBE checklist is best used in conjunction with this article (freely available on the Web sites of PLoS Medicine at http://www.plosmedicine.org/, Annals of Internal Medicine at http://www.annals.org/, and Epidemiology at http://www.epidem.com/). Information on the STROBE Initiative is available at www.strobe-statement.org.
