# Supplementary material for: Bruxism associated with short sleep duration in children with autism spectrum disorder: The Japan Environment and Children’s Study
Source: PLoS One. 2024 Dec 6;19(12):e0313024. doi: 10.1371/journal.pone.0313024 (PMC11623795; doi:10.1371/journal.pone.0313024)
Supplement: S1 Table — (DOCX) [file pone.0313024.s002.docx]

| **Table S1. Baseline association of sleep duration with the prevalence of PRB in 83,720 children** | | | | | | | | |
| --- | --- | --- | --- | --- | --- | --- | --- | --- |
|  | ***Control*** |  | | ***ASD*** | | |  | |
| Child's PRB | Absence, n (%) | Presence, n (%) | | Absence, n (%) | | | Presence, n (%) | |
|  | 76,823 (92.9) | 5,884 (7.1) | | 887 (87.6) | | | 126 (12.4) | |
| ***Infant's sleep duration at 1 month after delivery*** | | |  | |  |  | |  |
| ≤13 | 15,671 (91.5) | 1,458 (8.5) | | 229 (82.1) | | | 50 (17.9) | |
| >13 to 15 | 20,728 (92.4) | 1,713 (7.6) | | 263 (90.1) | | | 29 (9.9) | |
| >15 to 17 | 24,951 (93.4) | 1,772 (6.6) | | 251 (89.6) | | | 29 (10.4) | |
| >17 | 15,473 (94.3) | 941 (5.7) | | 143 (88.8) | | | 18 (11.2) | |
| ***at 6 months after delivery*** | | |  | |  |  | |  |
| ≤12 | 16,802 (92.3) | 1,407 (7.7) | | 233 (91.4) | | | 25 (8.6) | |
| >12 to 14 | 29,450 (92.8) | 2,283 (7.2) | | 324 (84.8) | | | 58 (15.2) | |
| >14 to 16 | 25,275 (93.3) | 1,817 (6.7) | | 266 (86.9) | | | 40 (13.1) | |
| >16 | 5,296 (93.4) | 377 (6.6) | | 64 (95.5) | | | 3 (4.5) | |
| ***at 12 months after delivery*** | | |  | |  |  | |  |
| ≤12 | 22,239 (92.6) | 1,772 (7.4) | | 274 (88.4) | | | 36 (11.6) | |
| >12 to 14 | 41,019 (93.0) | 3,084 (7.0) | | 467 (87.3) | | | 68 (12.7) | |
| >14 to 16 | 12,765 (93.0) | 964 (7.0) | | 139 (86.9) | | | 21 (13.1) | |
| >16 | 800 (92.5) | 64 (7.5) | | 7 (87.5) | | | 1 (12.5) | |
| ***at 18 months after delivery*** | | |  | |  |  | |  |
| ≤11 | 15,729 (92.5) | 1,282 (7.5) | | 208 (87.4) | | | 30 (12.6) | |
| >11 to 13 | 41,740 (93.1) | 3,079 (6.9) | | 452 (86.8) | | | 69 (13.2) | |
| >13 to 15 | 18,263 (92.7) | 1,439 (7.3) | | 211 (89.4) | | | 25 (10.6) | |
| >15 | 1,091 (92.8) | 84 (7.2) | | 16 (88.9) | | | 2 (11.1) | |
| ***at 36 months after delivery*** | | |  | |  |  | |  |
| ≤10 | 11,445 (93.1) | 855 (6.9) | | 164 (88.2) | | | 22 (11.8) | |
| >10 to 12 | 41,822 (92.8) | 3,242 (7.2) | | 486 (88.0) | | | 66 (12.0) | |
| >12 to 14 | 22,738 (93.0) | 1,705 (7.0) | | 227 (85.7) | | | 38 (14.3) | |
| >14 | 818 (90.9) | 82 (9.1) | | 10 (100.0) | | | 0 (0.0) | |

ASD = autism spectrum disorders; PRB = parent-reported bruxism.
