# Supplementary material for: Bruxism associated with short sleep duration in children with autism spectrum disorder: The Japan Environment and Children’s Study
Source: PLoS One. 2024 Dec 6;19(12):e0313024. doi: 10.1371/journal.pone.0313024 (PMC11623795; doi:10.1371/journal.pone.0313024)
Supplement: S2 Table — (DOCX) [file pone.0313024.s003.docx]

| **Table S2. Association of sleep duration with the prevalence of PRB in infants with or without ASD** | | | | | | |  |  |  |  |
| --- | --- | --- | --- | --- | --- | --- | --- | --- | --- | --- |
|  | **Presence (%)** | Crude | *p* value | Model 1^a^ | *p* value | Model 2^b^ | | | *p* value | |
| ***Sleep duration at 12 month after delivery (hrs)*** | | | | | | | |  |  |  |
| ***ASD*** |  |  |  |  |  |  | | |  | |
| ≤12 | 36 (11.6) | Ref |  | Ref |  | Ref | | |  | |
| >12 to 14 | 68 (12.7) | 1.13 (0.72-1.77) | 0.601 | 1.14 (0.72-1.79) | 0.583 | 1.17 (0.74-1.86) | | | 0.508 | |
| >14 to 16 | 21 (13.1) | 1.16 (0.64-2.11) | 0.619 | 1.17 (0.64-2.12) | 0.617 | 1.18 (0.64-2.20) | | | 0.596 | |
| >16 | 1 (12.5) | 1.27 (0.15-10.76) | 0.825 | 1.27 (0.15-10.76) | 0.826 | 1.40 (0.16-12.29) | | | 0.762 | |
| *OR per 1-hr increase* |  | 1.02 (0.91-1.14) | 0.707 | 1.02 (0.92-1.14) | 0.695 | 1.03 (0.92-1.15) | | | 0.651 | |
| ***non-ASD*** |  |  |  |  |  |  | | |  | |
| ≤12 | 1,772 (7.4) | Ref |  | Ref |  | Ref | | |  | |
| >12 to 14 | 3,084 (7.0) | 0.94 (0.88-1.01) | 0.080 | 0.94 (0.88-1.00) | 0.062 | 0.95 (0.89-1.02) | | | 0.147 | |
| >14 to 16 | 964 (7.0) | 0.95 (0.87-1.03) | 0.212 | 0.93 (0.86-1.02) | 0.106 | 0.95 (0.87-1.03) | | | 0.207 | |
| >16 | 64 (7.5) | 1.01 (0.77-1.33) | 0.941 | 0.98 (0.75-1.29) | 0.900 | 0.99 (0.75-1.29) | | | 0.911 | |
| *OR per 1-hr increase* |  | 0.98 (0.97-1.00) | 0.033 | 0.98 (0.97-1.00) | 0.015 | 0.99 (0.97-1.00) | | | 0.035 | |
| ***at 18 month after delivery (hrs)*** | | | | | | | |  |  |  |
| ***ASD*** |  |  |  |  |  |  | | |  | |
| ≤11 | 30 (12.6) | Ref |  | Ref |  | Ref | | |  | |
| >11 to 13 | 69 (13.2) | 1.09 (0.66-1.81) | 0.726 | 1.09 (0.66-1.81) | 0.733 | 1.14 (0.68-1.90) | | | 0.624 | |
| >13 to 15 | 25 (10.6) | 0.84 (0.44-1.62) | 0.596 | 0.82 (0.43-1.59) | 0.560 | 0.80 (0.41-1.56) | | | 0.516 | |
| >15 | 2 (11.1) | 0.73 (0.12-4.41) | 0.727 | 0.68 (0.11-4.17) | 0.679 | 0.77 (0.12-4.89) | | | 0.777 | |
| *OR per 1-hr increase* |  | 1.01 (0.91-1.12) | 0.831 | 1.01 (0.91-1.11) | 0.879 | 1.00 (0.90-1.11) | | | 0.999 | |
| ***non-ASD*** |  |  |  |  |  |  | | |  | |
| ≤11 | 1,282 (7.5) | Ref |  | Ref |  | Ref | | |  | |
| >11 to 13 | 3,079 (6.9) | 0.91 (0.84-0.97) | 0.005 | 0.91 (0.84-0.97) | 0.005 | 0.92 (0.86-0.99) | | | 0.017 | |
| >13 to 15 | 1,439 (7.3) | 0.97 (0.89-1.05) | 0.442 | 0.95 (0.87-1.04) | 0.242 | 0.96 (0.88-1.04) | | | 0.324 | |
| >15 | 84 (7.2) | 0.95 (0.74-1.21) | 0.665 | 0.92 (0.72-1.17) | 0.477 | 0.91 (0.71-1.16) | | | 0.442 | |
| *OR per 1-hr increase* |  | 0.99 (0.97-1.00) | 0.122 | 0.99 (0.97-1.00) | 0.054 | 0.99 (0.97-1.00) | | | 0.060 | |
| ***at 36 month after delivery (hrs)*** | | | | | | | |  |  |  |
| ***ASD*** |  |  |  |  |  |  | | |  | |
| ≤10 | 22 (11.8) | Ref |  | Ref |  | Ref | | |  | |
| >10 to 12 | 66 (12.0) | 1.01 (0.59-1.71) | 0.979 | 1.00 (0.59-1.70) | 0.995 | 1.02 (0.59-1.74) | | | 0.951 | |
| >12 to 14 | 38 (14.3) | 1.24 (0.69-2.23) | 0.477 | 1.23 (0.68-2.22) | 0.497 | 1.24 (0.68-2.26) | | | 0.486 | |
| >14 | 0 (0.0) | N/A | - | N/A | - | N/A | | | - | |
| *OR per 1-hr increase* |  | 1.03 (0.90-1.18) | 0.684 | 1.03 (0.90-1.18) | 0.695 | 1.03 (0.90-1.19) | | | 0.656 | |
| ***non-ASD*** |  |  |  |  |  |  | | |  | |
| ≤10 | 855 (6.9) | Ref |  | Ref |  | Ref | | |  | |
| >10 to 12 | 3,242 (7.2) | 1.04 (0.96-1.12) | 0.361 | 1.04 (0.96-1.13) | 0.327 | 1.05 (0.97-1.13) | | | 0.264 | |
| >12 to 14 | 1,705 (7.0) | 1.00 (0.92-1.10) | 0.927 | 0.99 (0.91-1.09) | 0.896 | 1.00 (0.91-1.09) | | | 0.930 | |
| >14 | 82 (9.1) | 1.34 (1.02-1.77) | 0.036 | 1.31 (0.99-1.72) | 0.058 | 1.28 (0.97-1.69) | | | 0.080 | |
| *OR per 1-hr increase* |  | 1.01 (0.99-1.02) | 0.508 | 1.00 (0.99-1.02) | 0.719 | 1.00 (0.99-1.02) | | | 0.711 | |
| ASD = autism spectrum disorders; PRB = parent-reported bruxism.  Odds ratio (95% confidence interval) (all such values). | | | | | | | | | |  |
| ^a^Adjusted for maternal age and infant's sex. | | | | | | | | | |  |
| ^b^Additionally adjusted for maternal factors (educational attainment, smoking and drinking habit), household income, and presence of sibling(s) in infants with Model 1. | | | | | | | | | |  |
