# Supplementary material for: Bruxism associated with short sleep duration in children with autism spectrum disorder: The Japan Environment and Children’s Study
Source: PLoS One. 2024 Dec 6;19(12):e0313024. doi: 10.1371/journal.pone.0313024 (PMC11623795; doi:10.1371/journal.pone.0313024)
Supplement: Attachment — Submitted filename: Response letter_PONE-D-24-13310.docx [file pone.0313024.s004.docx]

PONE-D-24-13310

Behavioral occurrence of bruxism in children with autism spectrum disorder: the Japan Environment and Children's Study (JECS). Masahiro Tsuchiya, Shinobu Tsuchiya, Haruki Momma, Ryoichi Nagatomi, Nobuo Yaegashi, Takahiro Arima, Chiharu Ota, Kaoru Igarashi, and the Japan Environment and Children’s Study Group.

Dear Dr. Edwin Ularte, the Editorial office in PLOS ONE

**We thank you for checking our manuscript and providing corrections, which have been helpful. We have revised the manuscript accordingly. Our point-by-point answers to the issues are as follows:**

(1)Please can you clarify whether you are reporting a prospective study or retrospective study. In the checklist you have mentioned the study as a prospective study but from the rest of the Methods it appears that this is a retrospective study. Please advise.

If you are reporting results from a retrospective study, then please provide the following information on the dates when data were accessed for research purposes in DD/MM/YYYY format. Please note that if this information is not included when your manuscript is resubmitted, it may be rejected

**We are grateful for your careful reading of our manuscript and useful comments. As shown in P4L63, P5L87-88, our results are from a dataset of the prospective birth cohort survey, the Japan Environment and Children’s study. In terms of your comment regarding the retrospective design, we guess that the subgroup analysis examined the association between infant’s sleeping duration and bruxism prevalence among children with ASD, which was prospectively diagnosed by medical doctors during the survey. In general, retrospective studies are conceived after some people have already developed the outcome of interest like disease/disorder. In contrast, in a prospective study like a dataset from the JECS, baseline information is collected from all participants in the same way using exactly the same questions and data collection methods for all subjects before disease/disorder developments. Thus, our study was prospective in design and carried out. Please refer to the following papers, which were designed as prospective cohort studies using the same dataset. (**Kikuchi K et al., Sleep quality and temperament in association with autism spectrum disorder among infants in Japan. Commun Med (Lond). 2023 Jun 16;3(1):82. doi: 10.1038/s43856-023-00314-9.; Kushima M, et al., Association Between Screen Time Exposure in Children at 1 Year of Age and Autism Spectrum Disorder at 3 Years of Age: The Japan Environment and Children's Study. JAMA Pediatr. 2022 Apr 1;176(4):384-391. doi: 10.1001/jamapediatrics.2021.5778). **Similarly, our manuscript includes the results of both healthy and ASD participants. Thus, to help readers understand that our study was designed as a prospective cohort study, we have revised the explanations about our model structure in the Methods (Page 11, Lines 188 – 202).**

**Additionally, regarding the date of the recruitment period for the JECS survey, our manuscript should avoid noting the start and end days because of the discrepancies among regions. The description of the days of the recruitment period is also restricted in the submission guideline of the JECS.**

(2)We kindly ask that you address the discrepancy in the ethics documentation. The individual Arata Yamazaki named as investigator on the ethics documents is not an author on the present submission. As we would generally expect the investigator named on the ethical approval to be an author on the submission, can you please provide an explanation for this situation?

**We are grateful for your careful reading of the manuscript and appropriate suggestions. As pointed out, I made a mistake in writing the name of “Shin Yamazaki”, who is included in the Appendix, as “Arata Yamazaki”. We again thank you for providing such a valuable check.**

(3) We noted a different IRB approval number (no. 100910001) in your online submission form which does not match with the approval document (2023-017). To proceed with the manuscript, we request you to provide the correct IRB document or update the correct IRB approval number in the online submission form. Please note that some fields in the submission form may reset with this change, so please go through your submission in full to ensure that all information is accurate and complete when resubmitting your manuscript.

**According to your suggestion, we have additionally attached an approval document for IRB (#100910001).**

Thank you for your attention to this request. We look forward to hearing from you.

**We wish to thank you for carefully reading our manuscript and providing comments and suggestions. These checks have helped improve the manuscript.**
