# Supplementary material for: Bruxism associated with short sleep duration in children with autism spectrum disorder: The Japan Environment and Children’s Study
Source: PLoS One. 2024 Dec 6;19(12):e0313024. doi: 10.1371/journal.pone.0313024 (PMC11623795; doi:10.1371/journal.pone.0313024)
Supplement: Attachment — Submitted filename: Responses_to_Reviewers_PONE-D-24-13310_R2.docx [file pone.0313024.s005.docx]

PONE-D-24-13310

Title: Bruxism associated with short sleep duration in children with autism spectrum disorder: the Japan Environment and Children's Study

Authors: Masahiro Tsuchiya, Shinobu Tsuchiya, Haruki Momma, Ryoichi Nagatomi, Nobuo Yaegashi, Takahiro Arima, Chiharu Ota, Kaoru Igarashi, and the Japan Environment and Children’s Study Group.

Dear Dr. Ayako Mochizuki, DDS, PhD.

Academic Editor in PLOS ONE

**We thank the editor and reviewers for repeatedly reading our manuscript and providing structural suggestions, which have helped improve the manuscript. We have considered these suggestions and have revised the manuscript accordingly. Our changes in the manuscript are shown in red. The point-by-point answers to the comments of the editor and reviewers are presented in bold below:**

Reviewer #1: My all comments have been addressed. The authors addressed all comments. I have no additional comments.

**We thank you for the thoughtful review of our manuscript and useful comments, which have helped us address the concerns raised with appropriate corrections and revisions.**

Reviewer #2:

Although the issues raised by Reviewers have been somewhat improved, a more appropriate explanation based on deeper understanding of bruxism is necessary.

**We thank you for carefully reviewing our manuscript and providing constructive suggestions, which have helped us in revising it appropriately. We have considered your suggestions related to the inappropriate definition of bruxism observed in the study participants. We have accordingly modified the relevant sentences in the text.**

1）”bruxism behavior”

------------The authors are responsible for accurately defining the extent and characteristics of the study participants.

The subjects in this study were bruxers that were determined by whether or not they were aware of their bruxism based on report by their parents. More specifically, the subjects in this study were child bruxers whose parents answered "Yes" to the question, "Does your child have bruxism?" at the ages of 2 and 4. First of all, this should be clearly stated and appropriate group name (i.e., abbreviation) for the subject group specific to this study should be defined. Bruxism is a diagnostic term, and the expression "bruxism behavior" is inappropriate because it leads readers to imagine a specific type of bruxism and confuses them. Use of "bruxism behavior" cannot be acceptable.

**As pointed out, the study participants were defined as those with parent-reported bruxism for readers’ ease of understanding. We believe that a consistent prevalence of parent-reported bruxism will improve research reproducibility when a parent-administered questionnaire is used for examining the prevalence of bruxism. Thus, we have included participants with bruxism observed by the parents at both time points (2 and 4 years of age) in the PRB group and have changed the term “bruxism behavior” to “parent-reported bruxism (PRB)” throughout the manuscript to avoid misunderstanding.**

**As we have added the above description in the Methods (page 7, line 121–page 8, line 128), we hope that the term, “parent-reported bruxism”, observed in the participants, and its abbreviation, “PRB”, would address your concerns. Please let us know if issues regarding the handling of this term and its abbreviation persist in the revised manuscript such that we can address them suitably.**

2） The reply from the author ”in terms of the definition for the local definition of “bruxism behavior” in this study, considering that consistent bruxism behavior increases a risk for dental tissue damage, participants with “bruxism behavior” observed at both time points (2 and 4 years of age) were categorized into the bruxism group throughout the manuscript.”

---------- Since basis and meaning of this theory are unclear, the sentences cannot be acceptable.

**We agree with you that the previous theory explaining the categorization method of parent-reported bruxism was not sufficient in terms of the basis and meaning of the theory. In accordance with your suggestion, we have revised the relevant points in the Methods (page 8, line 124–line 126).**

3）The question "Does your child have Bruxism?"

-----------What does the question "Does your child have Bruxism?" mean?

Does this question refer to, or include all of them? More information and explanation are needed in the Methods and Discussion parts regarding the meaning of this question wording.

**We understand your argument that the question “Does your child have bruxism?” was vague; however, unfortunately, the questionnaire did not include other information on bruxism observed by the parents. Therefore, our findings included all types and severities of bruxism, for example, grinding and clenching both in the and sleep/awake states, as you have pointed out. Thus, per your suggestion, we have provided further information and explanation in the Methods and Discussion (page 7, line 121–line 128; page 20, line 317–line 322).**

4）The reply from the author ”The bruxism behavior is distinguished into two circadian symptoms: sleep and awake (P4L51–L53; P20L316–L320), as pointed out by Reviewer #2. Regarding the main study limitation, the dataset did not include enough information on bruxism behavior observed during sleep/awake status. Although further studies with detailed estimation of bruxism behavior in children with ASD are warranted, it should be noted that Winocur and his colleagues previously observed a higher concordance of both sleep and awake bruxism at an interindividual level (Front Neurol 2019;10:443.). We added the above including the reference under limitations in the revised manuscript (P20L313–P21L322).”

----- -This reply does not address the reviewer's point that asked for clarification regarding the type of bruxism that was the subject of this study.

It should be more clearly stated in the Introduction and Methods parts whether the bruxism studied in this study was sleep bruxism or awake bruxism.

I cannot understand the intention behind suddenly citing only the paper by Winocur's group. The paper by Winocur's group assessed bruxism based only on simple questions. It is already known that questionnaires do not have a high accuracy rate for either SB or AB.

In particular, recent studies have shown that AB cannot be judged by questionnaire based on self-awareness (Ishimaru, J Prosthodont Res. 2024). In addition, a study that evaluated SB with higher reliability using electromyograms instead of questionnaires found no correlation between SB and AB at all (Mikami, J Prosthodont Res. 2024).

This paper should more clearly state its inability to distinguish between SB and AB as its limitations.

**We appreciate your suggestions. The type of bruxism (e.g. sleep or awake bruxism) must be clearly noted. However, the question used in the study could not definitively distinguish the bruxism type into sleep and awake states. Additionally, as suggested, the low accuracy rates for assessing sleep/awake bruxism in studies using questionnaires have been mentioned (****Ishimaru et al, J Prosthodont Res. 2024; Mikami et al, J Prosthodont Res. 2024). Following your suggestion, we have revised the relevant points in the Discussion, citing the above references (page 20, line 317–page 21, line 324).**

**We again thank the reviewer #2 for your careful reading of the manuscript and their appropriate opinions. We have considered your suggestions and revised the manuscript accordingly.**
